# Screening for early warning of psychological crisis and intervention in children aged 8–18 years with cancer: a historical controlled trial

**DOI:** 10.3389/fped.2023.1156185

**Published:** 2023-06-22

**Authors:** Lu Yu, Lin Mo, Yixuan Liu, Xiaoyan Huang

**Affiliations:** ^1^ Department of Outpatient, Children’s Hospital of Chongqing Medical University, Chongqing, China; ^2^ Chongqing Key Laboratory of Pediatrics, Ministry of Education Key Laboratory of Child Development and Disorders, National Clinical Research Center for Child Health and Disorders, China International Science and Technology Cooperation Base of Child Development and Critical Disorders, Chongqing, China; ^3^ Department of Pediatrics, Children's Hospital of Chongqing Medical University, Chongqing, China; ^4^ Department of Oncology, Children's Hospital of Chongqing Medical University, Chongqing, China

**Keywords:** children with cancer, psychological crisis, early warning, intervention, 8–18 years

## Abstract

**Background:**

Childhood cancer is becoming an emerging healthcare issue in mainland China. Extensive evidence in the literature has demonstrated that cancer and its treatment experience can cause psychological distress that can lead to developmental problems in children with cancer. This study aims to screen for early warning of psychological crisis in children aged 8–18 years with cancer, establish a model of early warning intervention for children with cancer, and explore its application effects.

**Methods:**

We recruited 345 children with cancer and aged 8–18 years as the study participants, of whom 173 children were selected as historical controls during the period between December 2019 and March 2020 and 172 children were selected as the intervention group during the period between July 2020 and October 2020. The routine nursing model was adopted for the control group, and the early warning and intervention model was applied for the intervention group. The early warning and intervention model consisted of four stages: (1) establishing a management team to assess the risk of psychological crisis, (2) developing a three-level early warning response mechanism, (3) developing specific response plans, and (4) creating an evaluation summary and optimization mode. The DASS-21 was used to evaluate the psychological status of children with cancer before and 3 months after the intervention.

**Results:**

The average age of the control group was 11.43 ± 2.39 years, with 58.96% boys and 61.27% diagnosed with leukemia. The average age of the intervention group was 11.62 ± 2.31 years, with 58.72% boys and 61.63% diagnosed with leukemia. There was a significant reduction in depressive symptoms (4.91 ± 3.98, *t* = 12.144, *P* < 0.05), anxiety symptoms (5.79 ± 4.34, *t* = 8.098, *P* < 0.05), and stress symptoms (6.98 ± 4.67, *t* = 11.22, *P* < 0.05) in the intervention group. The incidence rates of depression, anxiety, and stress were significantly lower in the intervention group (12.79%, 29.07%, and 5.23%, respectively) than in the control group (46.82%, 49.71%, and 27.17%, respectively) (all *P*'s < 0.05).

**Conclusions:**

Our study suggests that the early detection and timely management of psychological symptoms through a nursing intervention model can effectively reduce depressive, anxiety, and stress symptoms in Chinese children with cancer. Future work should include conducting qualitative interviews to understand the psychological experiences of children with cancer throughout their entire life cycle.

## Introduction

Cancer is a major disease that seriously threatens the physical and mental health of adolescents. Its incidence and survival rates are increasing year by year (1). In 2017, the number of new cancer cases among children and adolescents in the world was approximately 416,500 (2). Between 2018 and 2020, the number of new cancer cases among children and adolescents in China was approximately 121,145, with an average incidence rate of 126.48 million (3). With the development of, and advances in, medical technology, the 5-year survival rate for children with cancer has been increasing gradually, which is as high as 85% (4). This means that there is a growing number of children and their families facing a range of problems such as late complications (5), secondary tumors, poor behavior (6), and even death.

Adolescence is an important period for physical growth and mental development. When adolescents are diagnosed with serious conditions during this critical period, they may be more sensitive to physiological and emotional changes than adults, making them a high-risk group for psychological and mental health crises. Some studies have shown that the diagnosis and treatment of cancer make adolescents bear more psychological pressure than what is required, which leads to different psychological reactions during different stages of cancer (7–10). Excessive and persistent negative pressure increases the degree of negative emotions in children, and children tend to have negative cognition. The result is a weakening of their self-confidence to overcome the disease and lower treatment compliance, both of which reduce the effect of treatment and disease rehabilitation.

However, research has shown that children with cancer show both negative and positive psychological responses in their fight against cancer. According to the relevant literature, children possess a definite cultural knowledge base after the age of 8 years to have an initial understanding of society and their personal thoughts (11). As their age of education advances and their level of education increases, the autonomy, cognitive level, and judgment ability of younger minors improve significantly. An 8-year-old child can clearly discern which training class they want to attend and which teacher is more strict than others. They also show the ability to self-report the occurrence of events (12). Moreover, children are at a specific stage of transition to adulthood during this time and are prone to stress while fulfilling their mandatory bodily needs of growing up.

The psychological problems of children with cancer and the relevant intervention strategies to be adopted have attracted the attention of researchers. Many research groups have evaluated different types of psychological interventions for adolescents with cancer. A study of psychological crisis intervention models in foreign countries has been recorded as early as 1964 when American psychologist Gerald Caplan first proposed the theory of psychological crisis. He divided the formation and evolution process of psychological crisis into four stages. Later researchers proposed the development of ecological models, situational ecological models, psychological first aid, and seven-stage psychological crisis intervention models. Chinese researchers proposed a real-time reservation model, an online-to-offline model, and a cognitive regulatory model. These models were mainly applied to healthy children and therefore lacked research on early identification, warning, and intervention of psychological problems in adolescents with cancer. Therefore, this study will construct an early warning and intervention model for psychological crisis in children with cancer and explore its application effect.

## Materials and methods

### Study participants

Adolescents with cancer were recruited from the Children's Hospital of Chongqing Medical University. The inclusion criteria were as follows: (1) children aged 8–18 years (this age group has self-reporting capabilities); (2) children diagnosed with cancer by the standard guidelines for the diagnosis and treatment of childhood malignancies (13); and (3) children who provided informed consent. The exclusion criteria were as follows: (1) children suffering from severe organic or cognitive impairment and (2) children who were unable to understand the questionnaire. Exit criteria included the following: treatment was terminated during the study, and children or guardians were asked to withdraw midway during the study. The Institutional Review Board of Children's Hospital of Chongqing Medical University approved this study, File No.: (2018) Lun Li (Research) No. (1).

### Sample size calculation

Refer to the formula of sample size for controlled trials, $N1=N2=(Z_{\alpha }+Z_{\beta }{)}^{2}*2\alpha^{2}/\delta^{2}$, where *α* = 0.05 and *β* = 0.1. According to the bilateral test table, *Z*_0.05 _= 1.96 and *Z*_0.1 _= 1.282; after literature review (14), *σ* = 4.37 and *δ* = 1.64, and the sample size for each group was calculated as 150 cases. Considering a 20% loss rate in the samples, the final sample size was determined as 180 cases. During the study, eight cases in the control group terminated intervention due to changes in the condition, six cases in the intervention group terminated intervention, and one case withdrew midway. Finally, 173 patients recruited from December 2019 to March 2020 were selected as the control group, and 172 patients recruited from July 2020 to October 2020 were selected as the intervention group.

### Measurement tools

The Depression Anxiety Stress Scale (DASS-21) is a self-assessment scale and was translated into Chinese by Xu et al. (15); it contains three dimensions: depression, anxiety, and stress. Each dimension has seven entries, and each item is scored from 0 (never) to 3 (always). The DASS-21 has good test reliability in the adolescent group and can be used as both a norm-referenced test and a standard reference test when evaluating the negative emotions of adolescents (16). The author's previous research showed that the total Cronbach's alpha of the DASS-21 is 0.916, the subscale's alpha coefficient is 0.793–0.819, the Kaiser–Meyer–Olkin (KMO) value is 0.919 (>0.8), the Bartlett spherical test statistic is *χ*^2^ = 1,938.6993 (*P* < 0.001), and the scale has good reliability and validity. Based on the total score, there were five levels: (1) normal, depression (≤9), anxiety (≤7), and stress (≤14); (2) mildly abnormal, depression (10–13), anxiety (8–9), and stress (15–18); (3) moderately abnormal, depression (14–20), anxiety (10–14), and stress (19–25); (4) severe abnormal, depression (21–27), anxiety (15–19), and stress (26–33); and (5) extremely severe abnormal, depression (≥28), anxiety (≥20), and stress (≥34).

### Interventions

Patients in the control group received routine nursing care. Nurses received patients warmly when they were admitted, introduced them to the environment of the hospital, implemented the doctor's advice, communicated with them before the operation, listened to them, and took care of them.

The intervention group was based on routine nursing care and also implemented the early warning and intervention model of psychological crisis (hereafter called the early model). The early model includes four steps.

#### Establishing a management team

Two doctors formulated treatment plans and discussed patients' conditions with their family members. Of four nurses, one was responsible for coordination and quality control, and the others were responsible for implementing nursing interventions. Two psychological counselors developed the intervention plans, trained in psychological knowledge, and provided one-on-one counseling if necessary. One community worker mobilized the support resources of the community or school, and another organized volunteer activities. In addition, the team should receive training in terms of the characteristics of children's psychological and behavioral development and crisis intervention, skills of psychological counseling and program implementation, clinical application of cognitive behavioral therapy, training of comfortable nursing ability of pediatric nurse, and procedures and considerations of the intervention.

#### Developing a response plan with the three-level early warning

According to the scoring standard of the Depression Anxiety Stress Scale (DASS-21), the early warning of a psychological crisis in adolescents with cancer was divided into three levels (Table 1).

**Table 1:** The response plan with the three-level early warning for adolescents with cancer.

Level of early warning	Criteria for classification	Intervention
First level	The anxiety score ranges from 8 to 9, the depression score ranges from 10 to 13, or the stress score ranges from 15 to 18	1. Psychological intervention: cognitive behavior therapy, rational emotive therapy, game therapy, music therapy, reading intervention, etc. 2. Behavioral intervention: systematic desensitization, imitation learning, self-management, role-playing, self-confidence training, etc.
Second level	The anxiety score ranges from 10 to 19, the depression score ranges from 14 to 27, or the stress score ranges from 19 to 33	1. Intervention plan of first-level early warning. 2. Psychological counselors and community workers engage themselves in the implementation of the intervention and provide psychological support to pediatric patients
Third level	The anxiety score is greater than 19, the depression score is greater than 27, or the stress score is greater than 33	1. Intervention plans of first-level and second-level early warning. 2. The psychiatrist of the hospital holds consultations and transfers the outcomes to the relevant professional medical institutions in a timely manner.

##### Implementing intervention

Establish archives (the first week): general information, family education, medical insurance, and psychological status.

Physiological support (every week): doctors, nurses, and patients formulated plans and promoted orderly activities, providing patients with timely care, reducing physical injury in operation and side effects of drugs, and helping patients establish a regular and healthy lifestyle, for example, providing children with healthy food and ensuring that they get enough sleep.

Psychological support (every week): providing cognitive therapy through semistructured interviews, understanding patients' adverse cognition, and correcting through semantic analysis, imitation, or decentralization.

Behavioral therapy (every week): analyzing the frequency and severity of adverse behaviors of patients, and correcting through systematic desensitization, imitation learning, self-management, role-playing, and self-confidence training.

Social support (every week): holding a series of lectures on “caring for children with cancer”; holding theme activities on a weekly basis, such as music activities, painting activities, sharing stories, film appreciation, and knowledge contests; and establishing a network group, for example, online communication, release of disease-related knowledge, psychological counseling, and regular children education. For example, Xiao Ming (assumed name) told us that he would eventually die from cancer. Then, we corrected his misconception, gave him confidence in overcoming the disease, and asked the recovered children to share their stories.

(1) Evaluation and summary: after 3 months of early warning intervention, we assessed the current psychological status of the children with cancer by the DASS-21.

### Statistical analysis

SPSS Statistics 22.0 was used for statistical analysis. The measurement data are described as the mean [±standard deviation (SD)]. The count data are described as the frequency and percentage (%). The chi-square test was applied to compare the qualitative data. The Bonferroni method was used to correct the *P* value in pairwise comparison. *t*-test, paired *t*-test, and analysis of variance method were used for quantitative data conforming to normal distribution. All tests were two-sided, and statistical significance was defined as *P *< 0.05.

## Results

### Baseline characteristics

There were no significant differences in the baseline characteristics of the intervention and control groups. The study participants were young in age (55.07% were 8–12 years old) and comprised a higher percentage of males (58.84%), mainly lived in cities (66.96%), mostly taken care of by their parents (90%), and mostly diagnosed with leukemia (61.63%) and had medical insurance (74.2%). The caregiver's cultural level was relatively low (72.46% received secondary school education) (Table 2).

**Table 2:** General characteristics of the participants (*N* = 345).

Items	Control group (*n* = 173)		Intervention group (*n* = 172)		*Z/χ^2^*	*P*
	*n*	%	*n*	%		
Gender						
Boy	102	58.96	101	58.72	0.002	0.964
Girl	71	41.04	71	41.28		
Age (years)						
8–12	92	53.18	98	56.98	0.592	0.744
13–18	81	46.82	74	43.02		
Only child						
Yes	79	45.66	85	49.42	0.487	0.485
No	94	54.34	87	50.58		
Areas						
Urban	123	71.10	108	62.79	2.690	0.101
Rural	50	28.90	64	37.21		
Primary caregiver						
Parents	162	93.64	153	88.95	2.388	0.122
Other	11	6.36	19	11.05		
Education levels of caregivers						
Junior high school and below	134	77.46	116	67.44	1.877	0.060
Senior high school	28	16.18	47	27.33		
University and above	11	6.36	9	5.23		
Family education						
Democratic	58	33.53	42	24.42	6.37	0.082
Autocratic	54	31.21	73	42.44		
Doting	53	30.64	46	26.74		
Neglecting	8	4.62	11	6.40		
Major cancer diagnoses						
leukemia	106	61.27	106	61.63	0.005	0.944
Solid tumors	67	38.73	66	38.37		
Medical insurance						
Yes	121	69.94	135	78.49	3.335	0.189
No	52	30.06	37	21.51		
Follow-up						
Regular	150	86.71	140	81.40	1.81	0.147
Irregular	23	13.29	32	18.6		

### The levels of anxiety, depression, and stress

There was no significant difference in levels of depression, anxiety, and stress between the control group and the intervention group before the intervention. After the intervention, the levels of depression, anxiety, and stress in the intervention group (4.91 ± 3.98, 5.79 ± 4.34, 6.98 ± 4.67) were lower than those in the control group (9.91 ± 6.53, 8.79 ± 5.34, 12.95 ± 8.71), and the difference between the two groups was statistically significant (*P *< 0.05) (Table 3). In addition, the levels of depression, anxiety, and stress in the intervention group (4.91 ± 3.98, 5.79 ± 4.34, 6.98 ± 4.67) were greatly lower than those before the intervention (10.72 ± 7.13, 11.47 ± 6.95, 14.06 ± 8.21). Only anxiety levels (8.79 ± 5.34) in the control group were significantly lower than those before the intervention (11.68 ± 7.31) (Table 4).

**Table 3:** Comparisons of depression, anxiety, and stress scores in the two groups.

Group	Depression	Anxiety	Stress
Before the intervention			
Control group (*n* = 173)	10.72 ± 7.13	11.47 ± 6.95	14.06 ± 8.21
Intervention group (*n* = 172)	10.68 ± 7.43	11.68 ± 7.31	14.36 ± 7.33
*t*	0.051	−0.273	−0.358
*P*	0.959	0.785	0.721
After the intervention			
Control group (*n* = 173)	9.91 ± 6.53	8.79 ± 5.34	12.95 ± 8.71
Intervention group (*n* = 172)	4.91 ± 3.98	5.79 ± 4.34	6.98 ± 4.67
*t*	12.144	8.098	11.220
*P*	<0.05	<0.05	<0.05

**Table 4:** Comparisons of depression, anxiety, and stress scores in the self-control group.

Group	Depression	Anxiety	Stress
Control group (*n* = 173)			
Before the intervention	10.72 ± 7.13	11.47 ± 6.95	14.06 ± 8.21
After the intervention	9.91 ± 6.53	8.79 ± 5.34	12.95 ± 8.71
*t*	−1.634	−7.008	−1.827
*P*	0.104	<0.05	0.07
Intervention group (*n* = 172)			
Before the intervention	10.68 ± 7.43	11.68 ± 7.31	14.36 ± 7.33
After the intervention	4.91 ± 3.98	5.79 ± 4.34	6.98 ± 4.67
*t*	10.182	9.79	14.555
*P*	<0.05	<0.05	<0.05

### The detection rate of depression, anxiety, and stress

As shown in Table 5, there were no significant differences in the detection rate of depression, anxiety, and stress between the control group and the intervention group before the intervention. After the intervention, the detection rates of depression, anxiety, and stress in the intervention group (12.79%, 29.07%, and 5.23%) were lower than those in the control group (46.82%, 49.71%, and 27.17%), and the difference between the two groups was statistically significant (*P *< 0.05).

**Table 5:** Comparisons of the detection rates of depression, anxiety, and stress [*n* (%), *N* = 345].

Group	Depression	Anxiety	Stress
Before the intervention			
Control group (*n* = 173)	109 (63.01)	112 (64.74)	69 (39.88)
Intervention group (*n* = 172)	99 (57.56)	120 (69.77)	71 (41.28)
*χ*2	1.069	0.99	0.07
*P*	0.301	0.32	0.792
After the intervention			
Control group (*n* = 173)	81 (46.82)	86 (49.71)	47 (27.17)
Intervention group (*n* = 172)	22 (12.79)	50 (29.07)	9 (5.23)
*χ*2	46.083	14.536	28.928
*P*	<0.05	<0.05	<0.05

## Discussion

### Children aged 8–18 years with cancer have obvious psychological problems

Children aged 8–18 years with cancer have obvious psychological problems. In this study, it was found that the detection rates of depression, anxiety, and stress in the intervention group before the intervention were 57.56%, 69.77%, and 41.28%, respectively, which were higher than those of middle school students (29.0%, 51.0%, and 23.9%, respectively) (17). Our previous investigation involving 217 adolescents with cancer found that 91.7% of them had various degrees of psychological problems (18). The immaturity of children's own physical and psychological development is the subjective factor of their psychological problems. On the one hand, children aged 8–18 years are about to have or already have entered adolescence. Individual physiology develops rapidly before reaching maturity, and therefore, there are significant changes in their physiological, psychological, and social development. On the other hand, children with cancer also suffer from the physiological or psychological effects of cancer symptoms, treatment, and long-term complications (19–22). In addition, the negative influences of family, school, and society are important objective stimuli for psychological problems. The interaction between physiological, psychological, and social factors serves to heighten some contradictions that could be solved in the development process of physiology and psychology. If the characteristics of diversity, overlapping, and timeliness are present, the psychological problems of adolescents usually cannot be solved by an intervention.

### The early warning and intervention model effectively improves the psychological state of children with cancer

The research results showed that the score and the detection rate of depression, anxiety, and stress in the intervention group were all markedly lower than those in the control group after the intervention. This means the early model effectively improves the psychological state of children with cancer. Furthermore, in the intervention group, the scores for depression, anxiety, and stress were greatly different from those after the intervention. In contrast, the scores for depression and stress in the control group were not observably different from those after the intervention; only the difference in the score of anxiety was statistically significant. All these indicated that traditional nursing intervention could appropriately alleviate the anxiety of children with cancer, but had no obvious effect on depression and stress. The early model could provide timely preventive interventions by comprehensively assessing the psychological and social conditions of children, thereby effectively improving their psychological problems, consistent with reports by researchers such as Chen Jin (23–25). Research has confirmed that early warning intervention can effectively improve patients' psychological state (26) and enhance intervention effectiveness (27).

This study constructed an early warning and intervention model for psychological crisis in children with cancer. First, qualification setting and responsibility clarifying and a multidisciplinary team including doctors, nurses, psychological counselors, community workers, and volunteers were established. Teamwork can effectively improve the members' ability to solve problems. Second, we developed the response of early warning and included the appropriate psychological scale to scientifically formulate grading standards. We arranged the response personnel, formulated intervention programs, and implemented preventive intervention rigorously and in a systematic way. Preventive intervention was carried out to correct patients’ unreasonable beliefs or cognition; to help them establish peer, social, and information support system; and to prevent the occurrence of psychological crisis. In addition, the specific implementation details of the program were formulated. The intervention program was set up after considering the physical, psychological, social, and spiritual aspects and combined with various interventions. Online and offline interventions were carried out simultaneously, which compensated for the deficiencies of routine psychological intervention. After the intervention, evaluation, feedback analysis, and file improvement were conducted. On this account, personalized and precise warning interventions could be achieved, effectively preventing and reducing the occurrence of psychological crisis in children with cancer.

## Conclusions

Children with cancer have obvious mental health problems. The early warning and intervention model of psychological crisis contained three aspects, which were regular assessment of psychological crisis, screening for psychological crisis, and intervention of psychological crisis, which was conducive to early detection and intervention of psychological problems in children with cancer, thereby preventing the occurrence or reducing the severity of the psychological problems.

## Clinical implications

Clinicians and nurses should pay more attention to early diagnosis and intervention of children with cancer to improve the disease outcome. Future work should include conducting qualitative interviews to understand the psychological experiences of children with cancer throughout their entire life cycle.

## Study limitations

This study did not classify the patients' disease diagnosis. It is necessary to collect information on the diagnosis of children with cancer to research whether there are differences in different cancer types. In addition, this is a historical controlled trial and not a randomized clinical trial, and therefore, the presence of some confounding variables cannot be ruled out.

## Data Availability

The data analyzed in this study is subject to the following licenses/restrictions. The data that support the findings of this study are available on request from the corresponding author. The data are not publicly available due to privacy or ethical restrictions. Requests to access these datasets should be directed to molin999@126.com.
